# Determination of Dental Adhesive Composition throughout Solvent Drying and Polymerization Using ATR–FTIR Spectroscopy

**DOI:** 10.3390/polym13223886

**Published:** 2021-11-10

**Authors:** Arwa Almusa, António H. S. Delgado, Paul Ashley, Anne M. Young

**Affiliations:** 1 Division of Biomaterials and Tissue Engineering, UCL Eastman Dental Institute, London WC1X 8DA, UK; aldelgado@egasmoniz.edu.pt (A.H.S.D.); p.ashley@ucl.ac.uk (P.A.); anne.young@ucl.ac.uk (A.M.Y.); 2 Centro de Investigação Interdisciplinar Egas Moniz (CiiEM), Monte de Caparica, 2829-511 Almada, Portugal; 3 Unit of Pediatric Dentistry, Department of Craniofacial Growth and Development, UCL Eastman Dental Institute, London WC1X 8DA, UK

**Keywords:** ATR–FTIR, acetone, dental adhesive, dental polymers, degree of conversion, methacrylate polymerization, photopolymerization, polymerization kinetics

## Abstract

The of this study aim was to develop a rapid method to determine the chemical composition, solvent evaporation rates, and polymerization kinetics of dental adhesives. Single-component, acetone-containing adhesives One-Step (OS; Bisco, Anaheim, CA, USA), Optibond Universal (OU; Kerr, Brea, CA, USA), and G-Bond (GB; GC, Tokyo, Japan) were studied. Filler levels were determined gravimetrically. Monomers and solvents were quantified by comparing their pure Attenuated Total Reflectance-Fourier Transform Infra-Red (ATR–FTIR) spectra, summed in different ratios, with those of the adhesives. Spectral changes at 37 °C, throughout passive evaporation for 5 min, then polymerisation initiated by 20 s, and blue light emitting diode (LED) (600 mW/cm^2^) exposure (*n* = 3) were determined. Evaporation and polymerisation extent versus time and final changes were calculated using acetone (1360 cm^−1^) and methacrylate (1320 cm^−1^) peaks. OS, OU, and GB filler contents were 0, 9.6, and 5.3%. FTIR suggested OS and OU were Bis-GMA based, GB was urethane dimethacrylate (UDMA) based, and that each had a different diluent and acidic monomers and possible UDMA/acetone interactions. Furthermore, initial acetone percentages were all 40–50%. After 5 min drying, they were 0% for OS and OU but 10% for GB. Whilst OS had no water, that in OU declined from 18 to 10% and in GB from 25 to 20% upon drying. Evaporation extents were 50% of final levels at 23, 25, and 113 s for OS, OU, and GB, respectively. Polymerisation extents were all 50 and 80% of final levels before 10 and at 20 s of light exposure, respectively. Final monomer polymerisation levels were 68, 69, and 88% for OS, OU, and GB, respectively. An appreciation of initial and final adhesive chemistry is important for understanding the properties. The rates of evaporation and polymerisation provide indications of relative required drying and light cure times. UDMA/acetone interactions might explain the considerably greater drying time of GB.

## 1. Introduction

The bonding of restorative materials to dentine involves the replacement of the mineral hydroxyapatite phase with a resin-rich layer that is capable of enveloping the collagen network [1,2]. This hybrid layer is the singular most important mechanism for securing a bond to dentine [2,3]. For it to be stable, this layer should guarantee a continuous and insoluble link between the restorative material and the underlying substrate. Achieving this, however, is extremely difficult [4,5]. The formation of the hybrid layer and its stability depends upon the nature and chemistry of the adhesive system used.

Dental adhesives typically consist of a range of monomethacrylate and dimethacrylate monomers, solvents, and low levels of filler particles. In two-component adhesive systems, these can be split into a hydrophilic primer and a more viscous, hydrophobic bonding part. Alternatively, they can be supplied as a single component. Formulations may require a separate acid-etching step, be it self-etch or multimodal. Factors such as the hydrophobicity/hydrophilicity of monomers, presence of functional monomers, pH, chemical structure, filler load/type, and type of solvents are pivotal in the development of the hybrid layer [6,7,8,9].

Solvents in adhesive formulations are required to act as carriers for the resin monomer during infiltration in dentine’s water-rich environment, as well as to re-wet and expand the collagen network that may be partially collapsed after acid demineralization. They may additionally ionize acidic functional monomers and improve adhesive shelf-life [10]. Typically, the contemporary adhesive solvents are water, acetone, and ethanol [11]. Solvent composition impacts its evaporation likelihood, adhesive water-replacing ability, and bonding capacity [10]. Acetone-based adhesives are known to be more volatile and technique sensitive, with a much higher evaporation rate and decreased shelf-life, compared to alternatives [12]. Acetone has a water-chasing ability, allowing it to remove excess water from the surface of the tooth. However, due to its limited ability to ionize acidic monomers in the adhesive, self-etch and bond properties may be reduced. Solvent drying times are, clinically, highly relevant. If insufficient, residual solvents will strongly influence the state of dentine, polymerization of the mixture, network development, and final properties of the adhesive polymer and its ultimate bond strength [9,13,14,15].

Past research has shown that clinicians do not always comply with the recommended drying times set out by manufacturers [14,16]. Even when doing so, evidence has also shown that the recommended times may be under-representative of the true ideal time. Solvent molecules reduce the viscosity of the initial mixture, which directly impacts polymerization kinetics. Studies over the past years have confirmed the influence and impact of variable solvent evaporation times on properties such as the final degree of monomer conversion, ultimate tensile strength, bond strength, and water sorption and solubility [13,14,17,18,19]. Most of these studies have focused on the mass loss characterization upon drying, which does not provide information on the changes to the chemical composition of the formulation and resulting polymerization reaction [13,18,19].

Fourier-transform infra-red (FTIR) is recognized as a gold-standard method in research guidance, published by the Academy of Dental Materials (ADM), for the study of polymerization in dental materials [20,21,22]. As it is non-destructive, requires very small amounts of material, and can detect minimal changes in important functional groups, it is ideal to study the reaction kinetics of dental polymers. Sample sizes required for reaction kinetic studies are very small, due to the high reproducibility and small error variation in the technique [23]. The aim of this study was, therefore, to develop a simple, rapid FTIR technique that is able to simultaneously assess the drying rates, changes to composition, and subsequent polymerization kinetics using a minimal amount of sample. The null hypotheses are that, for three selected, acetone-based, commercial dental adhesives, the method developed is unable to detect differences in (1) composition before and after drying, (2) drying rates, and (3) polymerization rates and final levels.

## 2. Materials and Methods

### 2.1. Material

**Figure 1:**
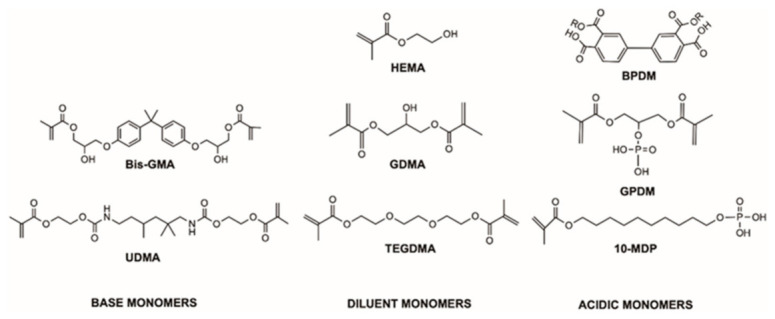
Chemical structure of monomers used in the commercial adhesives tested in this study, drawn with ChemBioDraw Professional (Perkin-Elmer, MA, USA) (BPDM is formed through reaction of anhydride groups on its benzene rings with the OH group on HEMA making *R* = C_2_H_4_MA, where MA is a methacrylate group).

Monomers, solvents, and fillers in the three commercial, single-component adhesives, investigated in this test: One-Step^®^ (Bisco Inc., Schaumburg, IL, USA), G-Bond (GC Corporation, Tokyo, Japan), and Optibond™ Universal (Kerr, Orange, CA, USA), are listed in Table 1. The chemical structure of the main monomers, found in these commercial adhesives and displayed in Figure 1, were drawn using the chemical software ChemBioDraw Professional 19.1.1 (Perkin-Elmer, Waltham, MA, USA).

### 2.2. Filler Isolation

Filler isolation was carried out to determine the total filler load and acquire spectra of fillers. To separate the fillers from the organic matrix, 10 mL of acetone was used to disperse 0.5 g of each adhesive. These acetone and adhesive levels were sufficient to enable easy removal of monomers and accurate determination of dried filler level using a four-figure balance, respectively. Agitation of the mixture for five minutes on a vortex mixer (Genie 2 model, Scientific Industries, Bohemia, New York, NY, USA) enabled complete dissolution/dispersion of the adhesive. Centrifugation at 4500 rpm for an hour (Sorvall Legend model, Thermo Fisher Scientific, Waltham, MA, USA) was sufficient to give clear supernatants. Following supernatant solvent and monomers removal, the sedimented filler was left to dry in a fume hood; 48 h of drying ensured the filler achieved a constant weight, which was used to determine its percentage of the adhesive (*n* = 3). Dried filler FTIR spectra were determined as below.

### 2.3. FTIR Spectra of Components and Adhesives

An FTIR spectrometer (FTIR Spectrum One, Perkin-Elmer, Beaconsfield, UK), coupled with an Attenuated Total Reflectance (ATR) golden gate accessory—Golden Gate ATR, (Specac, Orpington, UK), was used to obtain spectra of commercial adhesives, whilst drying and polymerising. Additionally, spectra of the main monomers and solvents, identified as potentially present, from information supplied by the manufacturers, were generated. The scanned monomers were Bis-GMA (Sigma-Aldrich, St. Gallen, Switzerland, LOT MKC L0376), HEMA (DMG, Hamburg, Germany, LOT 11220), GDMA (TCI, Tokyo, Japan, LOT 7RLBN TI), UDMA (DMG, Germany, Batch 97406), TEGDMA (DMG, Germany, Batch 88661), and 10-MDP (DM Healthcare, San Diego, CA, USA, LOT P01030). Solvents included deionized water, acetone (Sigma-Aldrich, Burlington, MA, USA), and ethanol (Sigma-Aldrich, Burlington, MA, USA). The separated filler required the use of the attenuated total reflectance (ATR) golden gate sapphire anvil to guarantee intimate contact with the diamond, while the liquids and adhesive mixtures did not. All spectra were acquired between 700 and 4000 cm^−1^, with a resolution of 8 cm^−1^ at 37 °C.

To semi-quantify the components in the commercial primers and adhesives before and after solvent evaporation, Microsoft Excel Tools v.16.35 (Microsoft, Redmond, WA, USA) was used to create model spectra. These were generated using the spectra of the pure monomers and solvents, as previously described in a method published by Delgado and Young (2021) [9]. With this method, the spectra of pure monomers, solvent, and fillers, known to be in the selected adhesives, were added together in different ratios, until all the main adhesive peaks and their relative heights could be accounted for. This model was based on the Beer–Lambert law and an approximately additive absorbance assumption. It assumed that the primer or adhesive absorbance (*A*), at a given wavenumber (*v*), was equal to the sum of absorbance of the individual monomers, solvents, and fillers, multiplied by their volume fractions, according to the following equation:

(1) $$A_{m,v}\;=\;\sum \left(A_{x,v}\;F_{x}\right)\;$$

where *A_m,v_* is the model (*m*) absorbance at wavenumber (*v*). *A_x,v_* is the solvent, monomer, or filler absorbance at the same wavenumber, and (*F_x_*) is their volume fraction. Ideally,

(2) $$\sum \left(F_{x}\right)\;=\;1$$

(3) $$\sum \left|\left(A_{m,v}-A_{a,v}\right)\right|\;=\;0\;$$

where *A_a,v_* is the absorbance of the actual adhesive mixture, the best fit was taken as that which gave the second equation equal to its minimum possible value. For the two components that were unavailable, BPDM and GPDM, it was assumed that their main peaks could be accounted for using a combination of the other monomers that have their main chemical groups.

### 2.4. Solvent Evaporation and Polymerization

To determine times required to evaporate acetone adhesives and how solvent retention and chemical composition may affect polymerization, kinetic studies were employed. The ATR surface temperature was set to 37 °C using a temperature controller (Specac Ltd., Orpington, UK). Spectrum TimeBase software (version 3.1.4, Perkin-Elmer, Waltham, MA, USA) was used to enable continuous generation of spectra, every 3 s. Experiment repetition number, *n*, for each adhesive (3.5 μL) of each adhesive was placed, in turn, on the ATR diamond using a micro syringe (SuperfleX™ Syringe, Sigma-Aldrich, Burlington, MA, USA). This was sufficient to provide a thin layer of the adhesive, comparable to that used clinically, which primarily just covered the ATR diamond. Solvent was allowed to evaporate, passively, for 5 min before 20 s light exposure using a light emitting diode (LED) curing unit Demi Plus (Kerr, Brea, CA, USA). This had an irradiance output of 600 mW/cm^2^, as measured with an analog radiometer Demetron Dental Curing Radiometer Model 100 (Demetron Research Corp., Danbury, CT, USA) and a wavelength of 450 nm to 470 nm. Spectra were collected during evaporation and for 5 min during/following cure, giving a total of 10 min acquisition time. Reasons for and clinical relevance of data collection and cure times are provided in the discussion.

Difference spectra for drying and polymerization were obtained by subtracting spectra at the start of each process from those at later times. These help to confirm what changes are taking place at different times and where maximum change is observed. Reaction extents, *R*, demonstrate how fast acetone evaporates and monomer concentrates during drying and polymerization rates following light exposure. These were calculated using Equation (4):

(4) $$R\;=\;\frac{A_{t}\;-\;A_{0}}{A_{f}\;-\;A_{0}}$$

*A* is the absorbance at 1360 cm^−1^, due to acetone, or at 1320 cm^−1^ for methacrylate monomers. Subscripts 0 or t indicate the time at or after, respectively, the start of drying or light exposure. For drying, both acetone and methacrylate peak absorbance were employed. During drying, whilst the acetone absorbance decreases, due to its evaporation, and the methacrylate peaks increase, due to their increasing concentration. For polymerization, only changes in the methacrylate absorbance were investigated.

Final absorbance values (subscript *f*), upon drying or curing, were estimated from the y intercept of linearly extrapolated data between 4–5 or 9–10 min, respectively, plotted versus inverse time (as inverse of zero is infinity). The time of half maximum change (when *R* = 0.5) for acetone evaporation and monomer concentrating (*t*_0.5_) for each sample was recorded and the standard deviation was calculated.

In order to calculate the degree of conversion at time t, the following Equation (5) was used:

(5) $$D_{C}\;\left(\%\right)=\left[1\;-\;\left(\frac{A_{t}}{A_{0}}\right)\right]\;*\;100$$

where *A*_0_ and *A_t_* are the C–O stretch absorbance at 1320 cm^−1^ above background level at 1345 cm^−1^ initially and at time t after start of polymerization [23]. The maximum degree of polymerization was calculated by finding the y axis intercept of the linear extrapolation of the late time *D_C_* data versus reciprocal of time.

### 2.5. Statistical Analysis

The variables *t*_0.5_ for solvent evaporation and final *D_C_* values were compared using the Statistical Package for the Social Sciences software (SPSS v.26.0 for Mac iOS (IBM Corporation, Armonk, NY, USA)) and one-way analysis of variance (ANOVA). Games–Howell post hoc test was used to overcome unequal variances. All analyses were carried out at a set significance level of 5%.

## 3. Results

### 3.1. FTIR Spectra of Pure Monomers, Solvents, Fillers, and Filler Fractions

**Figure 2:**
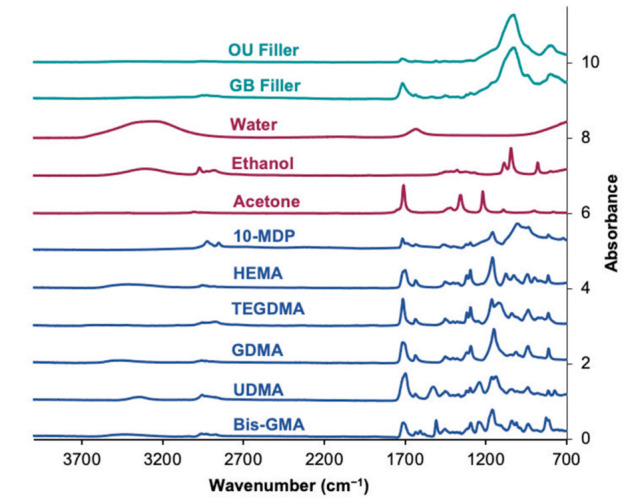
ATR–FTIR spectra of the individual fillers, solvents and monomers acquired to build the model fitting. Common and distinct peak assignments are provided in Table 2.

Spectra of the pure monomers and solvents are displayed in Figure 2. IR peak assignments are summarized in Table 2. Whilst the monomer spectra are dominated by peaks, due to the methacrylate groups, there are other characteristic peaks that help identify specific monomers. Spectra for the solvents are more distinctive and, therefore, easier to identify in mixtures.

**Figure 3:**
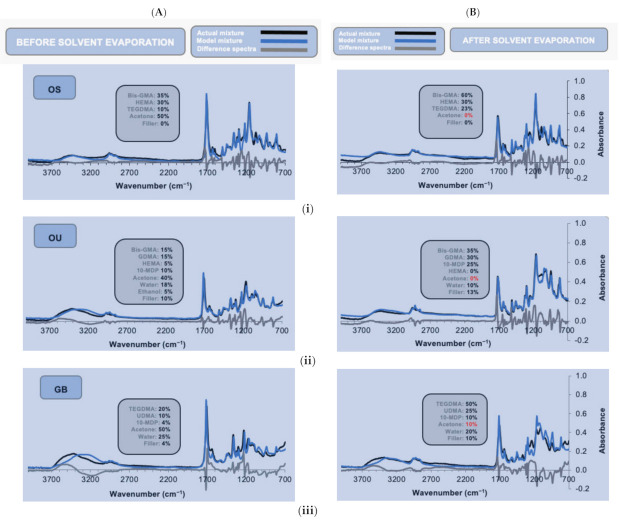
ATR–FTIR spectra of adhesives (**i**) OS, (**ii**) OU, and (**iii**) GB, featuring the actual mixtures (in black), model spectrum (in blue), and difference spectrum between the two spectra (in grey), (**A**) before and (**B**) after solvent evaporation.

The spectra for OU and GB sediments (also provided in Figure 3) are dominated by the strong Si-O peak at 1000 cm^−1^ but can also have some weak methacrylate peaks. Whilst OS gave no sediment, the mean sediment mass fractions and standard deviations for OU and GB were 9.6 ± 0.5% and 5.3 ± 0.5% (*n* = 3), respectively.

### 3.2. FTIR Compositional Modelling of Adhesives

Figure 3 illustrates the ATR–FTIR spectra of the single-component adhesive systems, the model fitting used to estimate pure chemical fractions within (see Section 2.3) and a comparison between before and after solvent evaporation.

For OS (Figure 3—OS), the initial spectrum was consistent with the formulation containing high levels of HEMA and Bis-GMA dispersed in acetone. HEMA and Bis-GMA, in conjunction with TEGDMA, were used to model the functional groups existent in BPDM, for which a model spectrum was unavailable. The differences around 3000 cm^−1^ [*v*(O–H)] could be a consequence of none of the model constituents having the COOH group of BPDM. Peaks and troughs in difference spectra at 1730 cm^−1^ [*v*(C=O)], 1255 cm^−1^ [*v*(C–O)], and 1170 cm^−1^ [*v*(C–O–C)] could also be a consequence of Bis-GMA with HEMA and TEGDMA not being able to account for all the functional groups of BPDM in the correct ratios. Alternatively, they may be caused by changes in O–H hydrogen bonding levels in mixtures, compared with the pure chemicals. Upon drying the acetone peaks almost completely disappeared. Other changes were consistent with approximate doubling in concentration of all the monomers except for HEMA, which remained unchanged.

Modelling of OU suggested it consisted of a Bis-GMA resin phase, with GDMA and lower levels of HEMA. Model fit was improved assuming a combination of GDMA and 10-MDP could model the methacrylate and phosphate groups, respectively of GPDM that was unavailable. Solvents identified were consistent with a co-mixture of acetone, water, and ethanol. Even after solvent evaporation, with monomer and filler concentration increasing, a good fit was obtained (Figure 3—OU). Whilst acetone and ethanol were lost upon evaporation, some water was still present after the 5 min drying period.

The model fit for GB (Figure 3—GB) was constructed using only the spectra of pure chemicals, identified from the SDS provided by the manufacturer and that of its filler. Modelling was consistent with a mixture of dimethacrylates (TEGDMA, UDMA), with 10-MDP as a functional monomer, dispersed in a co-mixture of acetone and water. Poor fit in some spectral regions and slow acetone evaporation could be explained by acetone, undergoing an acid catalyzed reversible reaction with the NH groups on UDMA to form an enamine and water. In this reaction, NH groups are replaced by a N–C=C moiety. This would explain the model over and underestimating the NH (1520 cm^−1^) and C=C (1640 cm^−1^) absorbance, respectively. As COO groups are attached to the NH groups in UDMA, the reaction could also account for multiple other intense C–O peak shifts within the fingerprint region. Formation of an intermediate carbinolamine (with an NCOH group) and variable hydrogen bonding could additionally cause peak shifts in the OH (around 3500 and 1100 cm^−1^) spectral regions. Good fit in the acetone 1358 cm^−1^ peak region, however, still enabled determination of the changes in acetone level upon drying.

### 3.3. Difference Spectra for Evaporation Versus Polymerisation

**Figure 4:**
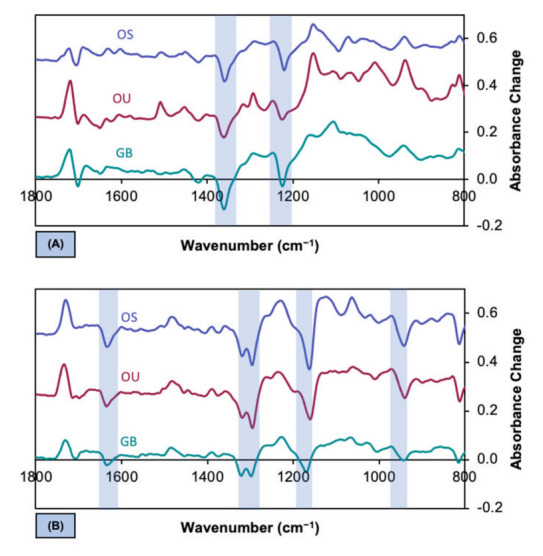
(**A**,**B**). Maximum difference in absorbance versus wavenumber (difference spectra) upon acetone evaporation (**A**) and upon polymerization (**B**) for OS, OU and GB. These were obtained by subtracting initial spectra from those at 5 min (**A**) and spectra at 5 min from those at 10 min (**B**), respectively. Blue bands indicate main acetone and monomer troughs in A and B arising, due to their loss upon evaporation and polymerization, respectively. Note—highlighted monomer troughs in B correspond with peaks in A due to solvent evaporation increasing monomer concentrations.

Figure 4A displays the evaporation difference spectra, obtained by subtracting the spectra at zero time from those at 5 min. Difference spectra, obtained by subtracting spectra before 5 min from the initial spectra, had similar peak and trough positions/relative levels but were of lower intensity. Troughs at 1220 and 1360 cm^−1^, in Figure 4A, are caused by acetone evaporation. Peaks in the difference spectra are caused by an increase in various different monomer concentrations.

Difference spectra for polymerization, obtained by subtraction of the 5 min spectra from those at any later time points, had peaks and troughs in identical positions, irrespective of adhesive. The final level of change, obtained by subtraction of the 10 min spectra from those at 5 min (Figure 4B), decreased in the order OS > OU > GB. Troughs, highlighted in Figure 4B, at 1640 cm^−1^ [*v*(C=C)] and at 1320, 1300, and 1160 [*v*(C–O)], are typical changes seen with methacrylate polymerization in dental restorative materials. The peaks observed at 1715 (C=O) and 1240, 1220, and 1150 (C–O) cm^−1^ are due to the vibration of these bonds in the methacrylate group varying upon polymerization of the adjacent C=C.

### 3.4. Compositional Changes upon Evaporation

Pure chemical and filler fractions in each formulation before and after 5 min of evaporation, determined through modelling, are compared in Table 3. In all cases the sums of fractions were slightly above 1. The moduli of differences were on average smaller for OU indicating a better fit.

All formulations had initial acetone fractions of 0.4–0.5, the majority of which was lost during the 5-min drying period. Whilst the small fraction of ethanol (0.05) in OB was also lost in this time, some water remained in both OU and GB. Initial and final solvent fractions were 0.5 and 0.0 for OS, respectively. These increased to 0.75 and 0.3 for GB, whilst OB had intermediate values. Model filler fractions in OU (0.1) and GB (0.4) increased 3.3- and 2.5-fold upon drying. HEMA fractions were either unchanged or reduced upon 5 min of drying. For all other larger, less volatile monomers, fractions increased between 1.7- and 2.5-fold upon drying.

**Figure 5:**
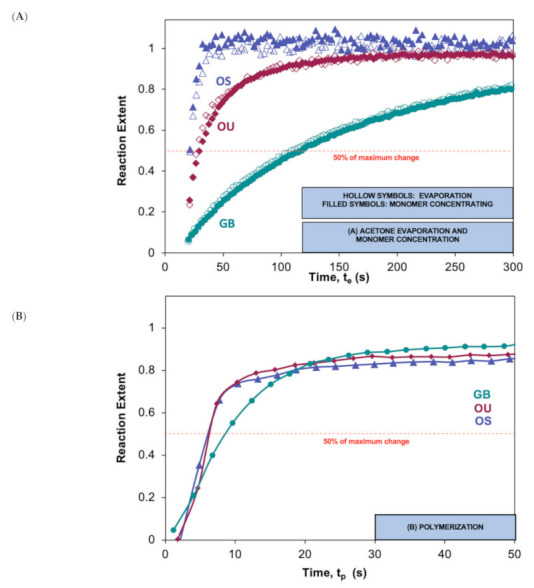
(**A**,**B**). Reaction extents for (**A**) acetone evaporation (hollow) and monomer concentrating (filled) versus time after start of experiment (t_e_) and (**B**) for methacrylate polymerization versus time after start of light exposure (t_p_) of the three tested adhesives.

For any given adhesive, acetone evaporation and monomer concentrating reaction extents (Equation (3)) were comparable at all times (Figure 5A), and *t*_0.5_ values were not significantly different (Table 4). This is as expected with consistent difference spectra. Significant differences (Welch’s ANOVA, *p* = 0.006 and 0.004) in *t*_0.5_, however, were observed between materials. Averaged results decreased in the order OS (23) > OU (25) > GB (113) (Figure 5A and Table 4).

### 3.5. Polymerization Extents and Final Conversions

Polymerization reaction extents (Equation (3)) rose rapidly upon light exposure and had *t*_0.5_ values all under 10 s (Figure 5B), whilst OS and OU reaction rates (gradient of reaction extent) had slowed substantially before 10 s that of GB remained high until 20 s. Consequently, whilst polymerization extents for OS and OU were higher, by 10 s, than for GB, all were close to 0.8 when the light was turned off at 20 s. Degrees of conversion (Equation (4)) at 20 s will, therefore, be 80% of the final conversions that are provided in Table 4 (i.e., 54, 55, and 70% for OS, OU, and GB, respectively). Final conversions were significantly lower for OS and OU than for GB (Welch’s ANOVA, *p* = 0.01).

## 4. Discussion

### 4.1. FTIR Method

This study employed a recently developed ATR–FTIR method to assess adhesive composition [9,21,24]. The technique is non-destructive, quick, simple, requires minimal sample amounts, and is less laborious, compared to Raman spectroscopy. Ease of sample access for curing and relatively fast rate of data acquisition enables continuous spectral acquisition before, during, and after light-curing [9,25]. The new method of prior solvent evaporation kinetics determination developed in this study, further extends the technique benefits.

Knowledge of material compositions is fundamental for providing an understanding of the material properties and how they should be used and adapted to each clinical scenario [9,26]. It allows researchers and clinicians to gain insight on setting, mechanical, and physical properties, as well as degradation processes (important for toxicity and longevity concerns). As the tested adhesives all had chemical differences that were readily detectable by FTIR before and after evaporation, the first null hypothesis was rejected.

Appreciation of relative evaporation rates is important because they may reduce shelf-life/stability and limit the penetration and self-etch properties of the adhesive if too fast. If slow and insufficient drying time is allowed, they can affect subsequent polymerization. Residual solvent may also cause adhesive plasticization and lower bond strength. Alternatively, phase separation, upon cure, can promote water-rich spaces, ultimately leading to biodegradation, catalyzed by hydrolysis, of the hybrid layer. This study showed that the FTIR method employed could easily detect significant differences in evaporation rates, so the second null hypothesis was rejected.

Polymerization rates are important for determining the recommended light-curing times, which can be material-dependent. Additionally, maximum cure is required for the polymer to achieve its optimal properties [14,27,28]. As 50% conversion was observed in under 10 s for all materials, the specific method employed was not ideal for detecting differences in early reaction kinetics. The final cure level for OS and OU were, however, significantly lower than that of GB. The third null hypothesis, that the FTIR method is incapable of differentiating polymerisation kinetics and final levels for the selected adhesives, was, therefore, partially rejected.

### 4.2. Material Selection

Clinicians often opt for simplified single-component adhesives. These require fewer time-consuming clinical steps and reduce the possibility of errors [3,29]. Whilst OS is used following tooth etch and rinse, OU can be applied with or without tooth etching and GB is self-etching.

The three adhesives selected for this study were all acetone based, which is a popular solvent found in single-component adhesive systems [11]. Unlike OS, the self-etching formulations OU and GB also contain water and filler. Furthermore, OS and OU are Bis-GMA, while GB is UDMA based. These differences enabled the determination as to whether the water and/or base monomer might be the likely cause of large differences in acetone evaporation and subsequent polymerization kinetics. Being a separate phase, however, fillers are less likely to be the cause of evaporation and polymerization differences.

In addition to the base monomer (Bis-GMA or UDMA), the adhesives can also contain high levels of smaller, more fluid, and/or hydrophilic diluent monomers that help to increase flow and polymerisation. These have hydrophilic hydroxyl (–OH) (e.g., in HEMA and GDMA) and/or flexible ethylene (–COC) (e.g., TEGDMA) chemical groups. Solvent interactions with the OH group could potentially also reduce evaporation rates.

The functional, acidic monomers with carboxylic (–COOH) (in BPDM) and phosphoric (H_2_PO_4_) acid groups (in GPDM and 10-MDP) may aid the mixing of hydrophobic and hydrophilic components. They may also promote etching or bonding to dentine by reacting with calcium in hydroxyapatite [26]. As they tend to accumulate at interfaces, they could potentially have complex effects on solvent evaporation and polymerisation kinetics. Manufacturer data sheets suggest that the chemical structures of the functional monomers in a given adhesive are similar to the other monomers employed, except for the acid groups. For example, BPDM in OS has aromatic groups, as in Bis-GMA, whilst GPDM in OU is similar to GDMA, except for the OH group being a phosphate moiety. Aliphatic 10-MDP might be more compatible with the non-aromatic UDMA and TEGDMA in GB. Monomer chemical similarities are likely to enhance their miscibility. It also means they can be modelled using other monomers and do not complicate spectra interpretation, which is beneficial for functional monomers with limited availability.

### 4.3. Initial FTIR Spectra of Adhesives

The assumption of additive absorbance was based on the classical Beer–Lambert law. As a limitation, it is important to understand that absorbance is only approximately additive and may be strongly affected by chemical interactions between the individual components [30]. Deviations from a simple Beer–Lambert law are particularly important in the case of strong oscillators, such as the inorganic filler particles. Furthermore, the intensity of solid peak spectra is determined by the pressure applied by the ATR anvil and particle size.

Despite this, the relative filler levels in the different materials obtained by FTIR were comparable with those seen by gravimetric studies and consistent with figures given by the manufacturers. Weak monomer peaks, found in the spectra of isolated fillers, could be related to monomer retention, due to lower acetone solubility or monomers with functional groups forming aggregates with the filler particles in the formulation. Additionally, inefficient removal of organosilanes in silane-treated fillers may also give organic peaks [31]. Furthermore, modelling using the filler, solvent, main base, and diluent monomer spectra generally gave a reasonable fit to the adhesive spectra. Slight improvements in the fitting could be achieved by assuming low levels of other molecules to account for peaks of unavailable acidic monomers.

Whilst manufacturer safety sheets suggested acetone levels were variable between formulations, the FTIR spectra showed that initial acetone levels were all comparable at 40–50%. Additionally, OU and GB both contained water at approximately half the acetone level. Gravimetric studies confirmed that, whilst OS is unfilled, OU has approximately double the filler content of GB.

For OS, the phenyl, ethyl, and methacrylate groups in BPDM were modelled using Bis-GMA with TEGDMA. BPDM is a monomer that is uncommonly found in other adhesive formulations [11]. The modelling suggests that the component ratios of OS are similar to Example 14, found in a patent of the manufacturer [32].

Conversely, OU had GPDM as an acidic monomer, which has been widely researched in previous studies [33]. According to the OU fitted model, strong aromatic peaks, consistent with Bis-GMA, were detected. This was not disclosed in the manufacturer’s safety data sheets. However, the component ratios found with OU’s modelling, including BisGMA, showed good correlation to Example 1, found in the patent published by the manufacturer [34]. Concealing some of the components of a dental product is not peculiar, as companies try to protect their trade secrets.

GB modelling suggests that UDMA and 10-MDP are the base and acidic monomers used in the adhesive mixture, respectively. This is in agreement with the safety data sheets divulged by the manufacturer. The modelling fit for GB, however, was not as satisfactory as the other tested adhesives in this study. This could be explained by the fact that UDMA, present in GB, is a secondary amine (NHR_2_) that is able to react with acetone to form an enamine. This acid-catalysed reaction, however, can easily be reversed through interaction with water.

### 4.4. Spectral Changes upon Drying and Polymerization

FTIR difference spectra, obtained by subtracting spectra at early times from those at later times, are useful for confirming that only one process is taking place during a given time period and for showing where the largest spectral change occurs. This is demonstrated through the lack of change in the positions of peaks and troughs with time. In this study, a large absorbance decrease at 1360 and increase at 1320 cm^−1^ was observed for all adhesives on drying. This is a consequence of the monomer coincidentally concentrating as the acetone evaporates. Absorbance at 1320 cm^−1^ exhibits a strong change in the opposite direction (i.e., decreases) upon polymerization. The difference spectra upon polymerization, seen in this work, are comparable with those found in other studies of polymerizing methacrylates [9,23,35].

### 4.5. Evaporation Kinetics

Ideally, in a clinical setting, all the acetone should evaporate before the light curing unit is turned on. Any residual acetone affects the viscosity and gel effect, which is critical in determining polymerization kinetics [36]. Previously, gravimetric studies have been employed to determine filler contents and assess solvent evaporation kinetics from adhesives [19]. For complex mixtures, unlike the new FTIR method in this work, this does not give which components are present or lost. This is crucial to enable better understanding of subsequent polymerization kinetics.

Based on preliminary trials, it was found that acetone displays a strong isolated peak, at 1360 cm^−1^, that can be monitored clearly using FTIR. Additionally, as this solvent has high volatility, solvent evaporation before polymerization is readily achieved. In preliminary studies, it was found that the use of a low, fixed adhesive volume and passive evaporation enabled reproducible evaporation kinetic results. Five minutes at 37 °C allowed enough time for the evaporation process to slow significantly and the acetone peak at 1360 cm^−1^ to virtually disappear for all adhesives. In the clinic, air drying is used to speed up evaporation. In preliminary studies, however, this was found to give much poorer reproducibility than passive evaporation. The latter was, therefore, used to enable differences between material evaporation rates to be more readily assessed. Additionally, a vacuum may be applied in the clinic to further enhance drying rates. The rates observed in this study, therefore, only provide the expected relative rates, rather than the actual results within the clinic. They do, however, clearly indicate which formulations will require greater drying to remove acetone.

Acetone evaporation rates were strongly dependent upon the different solvents/monomers’ combination. Slower evaporation may be a consequence of interactions between acetone and the other components. In the case of Bis-GMA-based adhesives, the early evaporation rates for OS were only slightly faster than OU. On the other hand, GB that has UDMA as a base monomer needed 5 times longer for the acetone to evaporate. Furthermore, the evaporation was incomplete, as 10% of acetone was still detected after 5 min of drying. A possible explanation is the formation of an unstable enamine product that manages to slow down the process and hinder acetone total evaporation.

Whilst almost all of the volatile solvents (acetone and ethanol) evaporated from the adhesives in 5 min, this was not the case for water, which is a less volatile solvent. Water removal from dental adhesives is of great importance for their optimum performance and the long-term stability of the restorative interface [5,37,38]. However, this can be very difficult to achieve. In the two adhesives with water in their formulations (OU and BG), water was still present, even after a prolonged period of drying (20 and 10%, respectively, of the remaining adhesive). This is in agreement with other studies that demonstrate that complete water elimination is not possible [13,39].

The ability of a solvent to evaporate from a mixture should be proportional to its partial vapour pressure. In ideal mixtures, according to Raoult’s law, partial vapour pressures equal mole fractions of a component times their pure vapour pressures. Water has a high boiling point (100 °C), in comparison with acetone (56 °C), and, hence, low vapor pressure, which hinders its total removal. At 37 °C, the vapour pressures of pure water and acetone are 47 and 375 mmHg, respectively [26]. As these solvents evaporate, their concentrations and partial vapour pressures will decline. This would explain the observed reduction in reaction extent versus time gradients and, therefore, evaporation rates at longer times.

For all tested adhesives, after 5 min of drying, the larger high boiling point monomers concentrated, due to solvents’ evaporation, doubling their concentration percentages in the final mixture. However, HEMA either was not detected in the final mixture, in the case of OU, or its percentage remained unchanged, in the case of OS. This means that some of it did evaporate because the volume of the evaporated adhesive is smaller. This would be a consequence of its moderate boiling point (213 °C).

### 4.6. Polymerisation Kinetics

In this study, adhesives were light cured for 20 s. Whilst 10 s light exposure of the adhesive is usually recommended, further light exposure occurs when the composite is applied. High cure during the first 10 s could help to ensure optimal mechanical properties and biocompatibility are achieved by the adhesive in the hybrid layer. Reaction over longer times could provide a mechanism for adhesive chemical bonding with monomers in the composite when it is placed.

Polymerization slows substantially when the light is turned off, due to continuing termination, but no further production of free radicals that enable propagation of the monomer chain reaction. Substantial reduction in rate also occurs when the glass transition temperature of the adhesive reaches that of the surroundings and the polymer changes from a rubber to glass. This temperature may be raised by the presence of residual solvents or smaller, more flexible monomers. Alternatively, with dimethacrylates, the reaction can slow at 50% conversion, as the process changes from predominantly linking of small molecules to crosslinking of polymer chains. This may be occurring with OS and OU, as the reaction slowed well before but continued for some time after; the light was turned off at approximately 50% conversion. Conversely, with GB, the reaction rate slowed predominately only after the light was turned off at 20 s, at which point the monomer conversion was over 70%. This suggests that this adhesive may be reliant upon the additional light exposure during composite placement to achieve its maximum conversion.

GB achieved the highest final extrapolated *D_C_*, while OS and OU were not significantly different from each other. With sufficient light exposure, the final degree of polymerization is largely related to the organic matrix composition (i.e., monomers) and system viscosity [40,41,42]. GB is a UDMA/TEGDMA based-system, less viscous and bulky, compared to Bis-GMA systems, with higher mobility facilitating chain encounters. These systems are known to polymerize more efficiently, in contrast to Bis-GMA-based composites [41,43].

### 4.7. Study Limitations

A limitation of the above FTIR method is the difficulty in the estimation of filler loads, whilst the use of the additional gravimetric method requires a lot more sample. Additionally, identifying chemicals that are present at low level (e.g., initiators and activators) is impossible. Lack of availability of all the individual chemicals present in the adhesive systems (e.g., BPDM and GPDM) can also cause modelling problems. Levels of these acidic components might be better determined, for example, through pH determination studies. Additionally, interactions between individual components in the heterogenous mixture results in peak shifts and changes to the mixture spectrum that are not being modelled [9]. In the future, a calibration curve, with known components of each system and at relevant ratios, might address this issue and provide stronger evidence for the causes of the interactions.

The method developed for evaporation rate determination was optimised for acetone-based solvents. With other main solvents, different peaks for analysis would need to be selected. A further issue with the method occurs if both evaporation and polymerisation occur simultaneously, as the changes in absorbance at 1320 cm^−1^ can cancel out. This invalidates the method employed for monomer conversion determination. Dealing with simultaneous evaporation and polymerization will require more complex methods of data interpretation. Additional improvements in the quantification and understanding of reaction rates might also be achieved by fitting the full reaction curves, instead of just obtaining the half-life. Data interpretation improvement studies are ongoing.

Caution is also required in interpreting the drying kinetics in vitro, which in the clinic could be accelerated by an air/water syringe. Furthermore, the method does not address how the presence of the tooth structure and water within might affect composition and reaction rates. Further studies (e.g., with Raman spectroscopy mapping) may also help to address how important these factors are.

## 5. Conclusions

Within the limitations of this study, ATR–FTIR can provide valuable information on compositional changes, in addition to the drying rates and subsequent polymerization kinetics of acetone-based dental adhesives. Whilst 50% of the acetone could evaporate from thin films of Bis-GMA-based OS and OU adhesives in under 25 s, acetone evaporation was much slower from the UDMA-based GB formulation. Spectra modelling suggested this may be due to acetone/UDMA interactions. Water in the OU and GB formulations was initially at lower levels than acetone but evaporated less. Following drying, 20 s light exposure gave final monomer conversions of 67, 68, and 88% for OS, OU, and GB, respectively. Conversions achieved 0.5 and 0.8 times these values in under 10 s and by 20 s of light exposure, respectively.

Further studies are needed to assess the different evaporation times that closely approximate clinical conditions, with the aid of active water drying. A variety of conditions could be simulated, such as water contamination or inadequate curing time, to assess how it affects the components levels in the adhesives to better assess their effects.

## Figures and Tables

**Table 1:** Commercial adhesive types and their main monomers, solvents, and fillers, according to data provided by manufacturers and safety data sheets (percentages shown were given in the safety datasheets). N/A indicates manufacturers did not disclose range.

Adhesive	Type/Batch	Composition
One-Step^®^ (Bisco Inc., Anaheim, CA, USA) OS	Two-step etch-and-rinse1900003092	Bis-GMA (10–30%), HEMA (10–30%), BPDM (10–30%) Solvent: Acetone (50–75%) Fillers: does not contain fillers
Optibond™ Universal (Kerr, Brea, CA, USA) OU	Universal/ Multimode 7020184	HEMA (1–10%), GDMA (1–10%), GPDM (1–10%), Solvent: Acetone (30–60%), water (N/A), ethanol (1–10%) Fillers: N/A
G-Bond (GC, Tokyo, Japan) GB	One-step self-etch 2010091	UDMA (5–10%), TEGDMA (5–10%), 10-MDP (2.5–5%) Solvent: Acetone (25–50%), water (15–25%) Fillers: Colloidal silica

Bis-GMA: bisphenol-a-glycidyl dimethacrylate; HEMA: hydroxy ethyl methacrylate; BPDM: biphenyl dimethacrylate; GDMA: glycidyl dimethacrylate; GPDM: glycerophosphate dimethacrylate; UDMA: urethane dimethacrylate; TEGDMA: triethylene glycol dimethacrylate; 10-MDP: 10-methacryloyloxydecyl dihydrogen phosphate.

**Table 2:** Peak assignments of the individual monomers (10-MDP, Bis-GMA, GDMA, HEMA, TEGDMA, and UDMA) or solvents (acetone, ethanol, and water) depicted in Figure 3. Left side shows common methacrylate peaks, transversal to all monomers used, whereas right side of the table depicts distinct peak assignments found in selected components.

Wavenumber (cm^−1^)	Methacrylate Peak Assignment	Wavenumber (cm^−1^)	Peak Assignment	Compound
2950–2850	C–H stretch	3400	O–H stretch	HEMA, Bis-GMA
1700–1715	C=O stretch	3460	O–H stretch	GDMA
1635–1640	C=C stretch	3300	O–H stretch/ N–H stretch	Water, Ethanol/ UDMA
1390, 1365	O–C–H bend	1610	Aromatic C=C stretch	Bis-GMA
1320, 1300	C–O stretch	1510	Aromatic C=C stretch	Bis-GMA
		1520	N–H bend	UDMA
		1358	CH_3_ bend	Acetone
		1235	C–O stretch	Bis-GMA, UDMA
		1220	C–C–C stretch	Acetone
		1145–1160	C–O stretch	10-MDP, MA
		1115 1075	C–O–C stretch C–OH stretch	TEGDMA HEMA
		1100/1050	C–OH stretch	Ethanol
		960	P–O stretch	10-MDP
		890	C–C–O stretch	HEMA
		880	C–C–O stretch	Ethanol

**Table 3:** Summary table of the fractions of pure chemical and filler spectra used to obtain the model spectra for the different adhesives initially (initial) and after acetone evaporation (at 5 min) but before cure. Deviation of the sum of fractions and modulus of differences between actual and model spectra from 1 and 0, respectively, demonstrate level of model fit. Values in brackets show where TEGDMA and 10-MDP have been used as models for BPDM and GPDM, respectively, for which pure chemicals were unavailable.

Components	Model Adhesive Chemical and Filler Fractions					
Monomers, Solvents or Fillers	OS Initial	OS 5 min	OU Initial	OU 5 min	GB Initial	GB 5 min
Bis-GMA	0.35	0.60	0.15	0.35	-	-
HEMA	0.30	0.30	0.05	-	-	-
GDMA	-	-	0.15	0.30	-	-
UDMA	-	-	-	-	0.10	0.20
TEGDMA (BPDM)	(0.10)	(0.23)	-	-	0.20	0.35
10-MDP (GPDM)	-	-	(0.10)	(0.25)	0.04	0.10
Total monomers	0.75	1.13	0.45	0.90	0.34	0.65
Acetone	0.50	0.00	0.40	0.00	0.50	0.10
Water	-	-	0.18	0.10	0.25	0.20
Ethanol	-	-	0.05	-	-	-
Total solvent	0.50	0.00	0.63	0.10	0.75	0.30
Filler	-	-	0.10	0.33	0.04	0.10
Sum of fractions	1.26	1.13	1.18	1.33	1.13	1.05
Sum of Modulus of difference	21	39	22	23	31	35
Background absorbance	0.01	−0.004	0.00	−0.004	0.003	0.03

**Table 4:** Times of half maximum solvent evaporation/methacrylate concentration change and final degrees of monomer conversion [standard errors] (*n* = 3)**.** Different capital letters in the same column indicate statistically significant differences (Games–Howell, *p* < 0.05). *D_C_* and *t*_0.5_ values were both significantly higher for GB than for the other two adhesives. (N.B. *t*_0.5_ for polymerization are not provided, as all were below 10 s and rates too fast to determine with high accuracy with the method employed).

Adhesive	*t*_0.5_ (s) for Solvent Evaporation Determined Using Peaks for Acetone [1360 cm^−1^] Methacrylate [1320 cm^−1^]		*D_C_* (%)
OS	23 [1.3] **^A^**	23 [1.3] **^A^**	67.5 [0.01] **^A^**
OU	25 [1.7] **^A^**	26 [2.7] **^A^**	68.8 [0.03] **^A^**
GB	112 [15.6] **^B^**	114 [13.3] **^B^**	88.3 [0.02] **^B^**

## Data Availability

The data presented in this study are available on request from the corresponding author.
